# Proenkephalin A 119–159 as an early biomarker of acute kidney injury in complex endovascular aortic repair: an explorative single-center cross-sectional study with the utilization of two measurement methods

**DOI:** 10.1186/s13741-025-00553-5

**Published:** 2025-07-04

**Authors:** Paulina Walczak-Wieteska, Konrad Zuzda, Jolanta Małyszko, Paweł Andruszkiewicz

**Affiliations:** 1 2nd Department of Anaesthesiology and Intensive Care, Medical University of Warsaw, Warsaw, Poland; 2 Department of Nephrology, Dialysis and Internal Medicine, Medical University of Warsaw, Warsaw, Poland; 3 Doctoral School of the Medical University of Warsaw, Warsaw, Poland

**Keywords:** Proenkephalin, PenKid, Biomarker, AKI, EVAR

## Abstract

**Background:**

Acute kidney injury (AKI) remains a significant complication following endovascular aneurysm repair (EVAR). Current diagnostic methods often detect kidney damage too late for effective intervention. This study evaluated proenkephalin A 119–159 as an early AKI biomarker after EVAR procedures, comparing point-of-care testing with the ELISA method.

**Methods:**

Between April 2022 and June 2024, 68 patients undergoing elective EVAR were enrolled. Blood samples were collected preoperatively and for three consecutive postoperative days.

**Results:**

AKI was diagnosed according to the KDIGO criteria, with proenkephalin A 119–159 measured via point-of-care (penKid) testing and laboratory ELISA method. AKI occurred in 18 patients (26.5%). penKid showed a superior diagnostic performance to ELISA, demonstrating moderate agreement with KDIGO criteria (Gwet’s *AC1* = 0.52, *p* < .001). While penKid exhibited high sensitivity (80% day 1), specificity was moderate (51%). AKI patients had significantly higher median penKid levels (96.47 pmol/L vs 63.01 ng/mL, *p* = .001), longer hospital stays (12 vs 9 days, *p* = .028), and lower 6-month survival (50% vs 88.1%, *p* = .006).

**Conclusions:**

penKid testing shows promise as an early AKI biomarker following EVAR procedures, particularly for identifying low-risk AKI patients. However, its moderate specificity suggests it should complement existing clinical assessment tools rather than replace them. These findings support incorporating penKid monitoring into structured AKI care bundles for improved perioperative kidney outcomes.

**Supplementary Information:**

The online version contains supplementary material available at 10.1186/s13741-025-00553-5.

## Background

Serum creatinine (SCr) has been the standard parameter for renal function (Losito 2023) for 90 years and has significant limitations. It is still an essential kidney parameter in perioperative and intensive care settings (Ronco et al. 2019; Bellomo et al. 2004). These flaws, including the delayed detection of renal dysfunction, have prompted the search for early biomarkers of acute kidney injury (AKI).

Proenkephalin A 119–159 (PENK) has emerged as a promising biomarker for AKI. This endogenous, monomeric peptide (Khorashadi et al. 2020; Beunders et al. 2017) maintains a stable concentration after collection, has a long in vivo half-life, and is independent of sex or age (Khorashadi et al. 2020). Studies have shown that elevated PENK plasma concentration correlates with the development of AKI in critical settings (Khorashadi et al. 2020; Ibrahim et al. 2022), with PENK preceding SCr increases in septic patients (Lin et al. 2023) and correlating with the severity of AKI (Caironi et al. 2018).

Postoperative AKI often complicates endovascular aneurysm repair (EVAR), with incidence rates up to 50% (Cucuruz et al. 2020; Saratzis et al. 2015; Saratzis et al. 2016). Risk factors include chronic kidney disease, atherosclerotic disease, and limited cardiovascular reserve (Saratzis et al. 2016). While PENK has been studied in various clinical settings, limited research exists (Gombert et al. 2020; Doukas et al. 2024; Walczak-Wieteska et al. 2024) on its use in vascular surgery. Our study is the first to compare the bedside PENK test with the ELISA laboratory method (PENK/ELISA) in EVAR procedures. We aimed to evaluate the PENK’s utility as a predictive AKI biomarker in a perioperative setting and the incidence of sAKI in the EVAR population.

## Methods

This single-center, prospective, observational, cross-sectional study was conducted between April 2022 and June 2024 at the Central Teaching Hospital, Medical University of Warsaw. We enrolled 68 patients undergoing elective fenestrated or branched EVAR procedures. The study received ethics committee approval (8/KBL/OIL/2019 and 53/KBL/OIL/2022), and all participants provided written informed consent.

Blood samples were collected from arterial lines preoperatively and for three consecutive postoperative days. For PENK/ELISA measurement, plasma was collected in EDTA tubes, centrifuged, and stored at − 80 °C. The generic PENK ELISA kit was used strictly following manufacturer guidelines. The assay sensitivity was 0.041 ng/mL (1.33 pmol/L), range 0.05–10 ng/mL (1.62–324.81 pmol/L), and intra-assay precision < 9%. AKI was diagnosed according to the *Kidney Disease: Improving Global Outcomes *(KDIGO) 2012 criteria (Khwaja 2012). Following analogical studies, we defined subclinical AKI (sAKI) as a condition where penKid concentrations exceeded 80 pmol/L in the absence of an immediate rise in serum creatinine or fall in urine output that would meet standard KDIGO criteria (Dépret et al. 2020; Breidthardt et al. 2018). Point-of-care penKid was measured immediately with IB10 sphingotest®*penKid® assay* designed as a lateral flow test by SphingoTec GmbH, Hennigsdorf, Germany. The lowest detection limit of the immunoassay was 50 pmol/L.

We collected perioperative data, including demographics, medical history, and laboratory tests, and calculated the Sequential Organ Failure Assessment (SOFA) and the Simplified Acute Physiology Score (SAPS) scores on surgery day. Patients were monitored for complications, including AKI, hemodynamic instability, the administration of vasopressors and blood products, and mortality through 6-month post-procedure. The current study set a significance level of *α* = 0.05, permitting a 5% likelihood of committing a type I error. Analyses were conducted using the R statistical language (version 4.3.3; R Core Team, 2024) on Windows 11 64 bit (build 22,631), using the publicly available packages (Appendix 1).

## Results

The study involved 68 adult patients who underwent elective EVAR procedures. Two patients were excluded due to a single SCr measurement (before and after). The AKI occurrence referred to a cumulative occurrence for 3 days postoperative. Throughout observation, from the preoperative day to the third postoperative day, AKI occurred in 18 patients (26.5%) out of 66 following the AKI KDIGO criteria (Khwaja 2012).

The median age of the overall cohort (Table 1) was 70.5 years (*IQR*: 62.75–75.25). Patients in the AKI group were notably older, with a median age of 74.5 years (69.5–81.0), compared to 69.0 years (61.0–73.0) in the non-AKI group (*p* = 0.010). The cohort included 25 women (36.8%) and 43 men (63.2%). A higher proportion of men (77.8%) was observed in the AKI group compared to the non-AKI group (58.3%), although this difference did not reach statistical significance (*p* = 0.144). Regarding body mass index (BMI), the median BMI for the cohort was 26.08 kg/m^2^ (24.31–29.58). There was no statistically significant difference in BMI between the AKI and non-AKI groups. SCr levels at baseline were significantly higher in the AKI group, with a median value of 1.39 mg/dL compared to 1.01 mg/dL in the non-AKI group (*p* < 0.001).

**Table 1:** Sociodemographic profile of the patient cohort, stratified by AKI occurrence

Characteristic	*N*	Overall cohort^a^	AKI occurrence		*p*^c^
			Yes *n*_1_ = 18^a^	No *n*_2_ = 48^a^	
Age, y	66	70.50 (62.75, 75.25)	74.50 (69.50, 81.00)	69.00 (61.00, 73.00)	.010
Gender	66				0.144^d^
Female		24 (36.36%)^b^	4 (22.22%)^b^	20 (41.67%)^b^	
Male		42 (63.67%)^b^	14 (77.78%)^b^	28 (58.33%)^b^	
BMI, kg/m^2^	66	26.08 (24.31, 29.58)	26.25 (24.98, 30.78)	26.27 (24.31, 29.58)	0.751

^a^Median (Q1, Q3)

^b^*n* (%)

^c^Wilcoxon rank-sum test

^d^Pearson’s chi-squared test

Among the studied severity scores (Table 2), there is a notable distinction between the groups regarding the SAPS and SOFA scores. The SAPS score was elevated in the AKI group (median 6.49 vs. 3.72, *p* = 0.008), indicating that patients with AKI exhibited more severe physiological derangements. The SOFA score also differed significantly between the two groups, with a median of 6.40 in the non-AKI group and a much higher interquartile range in the AKI group, peaking at 20.20 (*p* = 0.048). The need for transfusions was also markedly higher in the AKI group, with 83.33% of AKI patients receiving transfusions postoperatively, compared to 33.33% in the non-AKI group (*p* < 0.001).

**Table 2:** Characteristics of perioperative critical care and outcome parameters of the study cohort, stratified by AKI occurrence

*Characteristic*	*N*	*Overall cohort*^*a*^	*AKI occurrence*		*p*^c^
			*Yes* *n*_*1*_ = *18*^a^	*No* *n*_*2*_ = *48*^a^	
SAPS %	66	3.95 (1.74, 5.82)	6.49 (3.13, 8.77)	3.72 (1.69, 4.67)	.008
SOFA %	66	6.40 (6.40, 9.85)	6.40 (6.40, 20.20)	6.40 (4.80, 6.40)	.048
Transfusion	66	31 (45.59%)^b^	15 (83.33%)^b^	16 (33.33%)^b^	<.001^e^
Duration of hospitalization, days	66	9.00 (7.00, 11.25)	12.00 (8.25, 18.25)	9.00 (7.00, 11.00)	.028
Decease during hospitalization	66	2 (2.94%)^b^	2 (11.11%)^b^	0 (0)^b^	.071^d^
Survival, Follow-Up 6 months	56	44 (78.57%)^b^	7 (50.00%)^b^	37 (88.10%)^b^	.006^e^

^a^Median (Q1, Q3)

^b^*n* (%)

^c^Wilcoxon rank-sum test

^d^Fisher’s exact test

^e^Pearson’s chi-squared test

Regarding clinical outcomes, the duration of hospitalization was significantly longer in the AKI group, with a median of 12.0 days compared to 9.0 days in the non-AKI group (*p* = 0.028). Importantly, inhospital mortality was higher among patients with AKI, with 11.11% of AKI deceased patients during hospitalization compared to no deaths in the non-AKI group. However, this difference did not reach statistical significance (*p* = 0.071). The 6-month survival rate was significantly lower in the AKI group, with only 50.00% surviving at follow-up compared to 88.10% in the non-AKI group (*p* = 0.006).

Table 3 illustrates the longitudinal trends of these markers. It summarizes the mean concentrations of the penKid and PENK for the overall cohort, stratified by AKI occurrence.

**Table 3:** Mean concentrations of penKid and PENK over time for the overall cohort were stratified by AKI occurrence according to KDIGO criteria

*Characteristic*	*N*	*Overall cohort*	*AKI occurrence*		*p*^*b*^
			*Yes* *n*_*1*_ = *18*^a^	*No* *n*_*2*_ = *48*^a^	
*Baseline — postoperative day 3*					
penKid, pmol/L	66	69.83 (56.19, 93.55)	96.47 (74.38, 182.54)	63.01 (54.89, 78.60)	.001
PENK/ELISA pmol/L	66	44.50 (34.43, 72.76)	55.54 (40.28, 84.13)	40.93 (32.81, 61.39)	.057

^a^Median (Q1, Q3)

^b^Wilcoxon rank-sum test

The penKid exhibited a highly significant difference between groups, with a median = 96.47 pmol/L (*IQR*: 74.38, 182.54) in the AKI group compared to median = 63.01 pmol/L (54.89, 78.60) in the non-AKI group (*p* = 0.001). In contrast, PENK/ELISA approached significance (*p* = 0.057), suggesting a potentially weaker association with AKI. The correlation between penKid and PENK/ELISA was also assessed with rho =  − 0.06, indicating little to no association between these two markers.

The results of the agreement between AKI diagnosis by KDIGO criteria and penKid and PENK/ELISA were assessed. Gwet’s AC1 provided a more stable estimate than other metrics, particularly in the presence of imbalanced categories. penKid with a Gwet’s *AC1* = 0.52 (*p* < 0.001) with a confidence interval (*CI* 95%: 0.31–0.73) demonstrated moderate agreement with the KDIGO criteria (Table 4). Conversely, PENK/ELISA showed minimal agreement with the KDIGO criteria (*AC1* = 0.10, *p* = 0.428).

**Table 4:** The agreement results between AKI diagnosis by KDIGO criteria and penKid and PENK

*AKI biomarker*	*Gwet’s AC1*	*SE*	*CI 95%*	*p*
penKid	0.52	0.11	0.31–0.73	** <.001**
PENK/ELISA	0.10	0.12	− 0.15–0.34	0.428

### Subclinical AKI criterion

penKid concentrations exceeding 80 pmol/L were interpreted as indicative of sAKI. To further evaluate the agreement between the predicted and observed classifications, Gwet’s AC1 was calculated as a chance-corrected measure. McNemar’s test determined the difference between cases where the penKid prediction did not match the observed AKI status — at each time point.

Additional metrics included prevalence, defined as the proportion of patients diagnosed with AKI at each postoperative time point based on creatinine levels, and the detection rate, which indicated the proportion of the total sample correctly identified as AKI cases by penKid. The detection prevalence represented the proportion of the total sample predicted to have AKI by proenkephalin, regardless of the diagnosis.

The overall accuracy of penKid for predicting sAKI varied slightly across the postoperative time points (Table 5). On day 1, the accuracy was 0.55 (95% *CI*: 0.43–0.68), increasing marginally to 0.57 (0.42–0.70) on day 2, and then returning to 0.55 (0.36–0.73) on day 3.

**Table 5:** Performance metrics and the predictive capability of penKid levels (the predicted variable) for predicting the occurrence of AKI based on creatinine levels (the observed variable) at various postoperative time points

*Performance metric*	*Postoperative time point*		
	*Day1* *(n* = *65)*	*Day 2* *(n* = *53)*	*Day 3* *(n* = *31)*
Accuracy (*CI 95%*)	0.55 (0.43–0.68)	0.57 (0.42–0.70)	0.55 (0.36–0.73)
No information rate (NIR)	0.85	0.77	0.61
*p*-value [accuracy > NIR]	1.000	1.000	0.82
Gwet AC1 (*CI 95%*)	0.18 (− 0.08–0.45)	0.17 (− 0.11–0.46)	0.10 (− 0.27–0.46)
McNemar’s test *p*-value	< 0.001	< 0.001	0.061
Sensitivity (AKI occurrence as positive class)	0.80	0.75	0.75
Specificity	0.51	0.51	0.42
Positive pred value	0.23	0.31	0.45
Negative prediction value	0.93	0.88	0.73
Prevalence	0.15	0.23	0.39
Detection rate	0.12	0.17	0.29
Detection prevalence	0.54	0.55	0.65
Balanced accuracy	0.65	0.63	0.59

*n* number of complete pairs of data, *CI 95%* confidence interval 95%

However, the no information rate (NIR) was higher than the observed accuracy at each time point, with values of 0.85 on day 1, 0.77 on day 2, and 0.61 on day 3.

The sensitivity of penKid — its ability to correctly identify patients who developed AKI, presented with values of 0.80 on day 1 and 0.75 on days 2 and 3. The specificity was moderate to low, remaining stable at 0.51 on days 1 and 2 and dropping to 0.42 on day 3. The positive predictive value (PPV) reflected the likelihood that a patient predicted to have sAKI develops AKI, with values of 0.23 on day 1, 0.31 on day 2, and 0.45 on day 3.

Gwet’s AC1, a chance-corrected measure of agreement, demonstrated low values across all time points, with 0.18 (95% *CI*: − 0.08 to 0.45) on day 1, 0.17 (− 0.11–0.46) on day 2, and 0.10 (− 0.27–0.46) on day 3.

The prevalence of AKI increased as the postoperative period progressed, from 0.15 on day 1 to 0.23 on day 2 and 0.39 on day 3. This trend was consistent with clinical expectations, as AKI often develops in response to perioperative stress and physiological changes. The detection rate, which reflected the proportion of the total sample correctly identified as AKI cases by penKid, followed a similar pattern, increasing from 0.12 on day 1 to 0.17 on day 2 and 0.29 on day 3.

## Discussion

### Patient’s cohort

In this single-center, prospective, cross-sectional, observational study involving a cohort of unselected patients following complex EVAR, the incidence of AKI was observed to be 26.5%. This occurrence was elevated compared to recently published research findings, reporting incidence rates up to 18% (Villa 2024; Finnesgard et al. 2023). These differences were attributed to several factors, including the type of surgery (elective versus emergency) and specific patient characteristics. The sociodemographic profile data suggested that older age may be a significant risk factor for AKI in this patient population (Tallgren et al. 2007; Saratzis et al. 2020). However, this is contrary to previously published data in the EVAR group (Castagno et al. 2016). BMI did not demonstrate a strong association with the occurrence of AKI.

Patients with AKI had higher SAPS and SOFA scores. These findings reinforce the notion that AKI is associated with greater systemic organ dysfunction in the postoperative period. The patients who developed AKI were more likely to experience significant perioperative blood loss or other complications requiring transfusion, which could have contributed to or exacerbated renal injury due to hemodynamic instability (Statius 2017) or ischemia–reperfusion injury (Tiwari and Kapitsinou 2022). The prolonged hospitalization period reflects the more complicated recovery trajectory for patients with AKI, likely due to the need for advanced monitoring and supportive care. This suggests also that patients who developed AKI were in a more critical condition postoperatively.

### PenKid robustness

The significant difference in median penKid concentration between AKI and non-AKI patients suggests that penKid is a robust marker for the early detection of AKI and may have substantial clinical utility in identifying patients developing AKI, especially at low risk. In contrast, PENK/ELISA approached lower significance, suggesting a potentially weaker association with AKI criteria. While this marker may still hold some value, its discriminative ability appears less robust.

### Detection of AKI

penKid with a Gwet’s *AC1* = 0.52 demonstrated moderate agreement with the KDIGO criteria, indicating its potential utility as a reliable marker in clinical practice. Its relatively narrow confidence interval (*CI* 95%: 0.31–0.73) further supports its stability as a diagnostic tool for AKI. Conversely, PENK/ELISA showed minimal agreement with the KDIGO criteria with a confidence interval that spans negative values, suggesting that this biomarker may not be a reliable indicator of AKI compared to the KDIGO standard.

The penKid’s sensitivity and ability to correctly identify patients who developed AKI were consistently high across all time points. This indicates that penKid was reliable in detecting AKI when it occurred, making it a valuable tool for identifying at-risk patients. However, the specificity was moderate to low, remaining stable at 0.51 on days 1 and 2 but dropping to 0.42 on day 3. This suggests that while penKid is sensitive to AKI occurrence, its ability to correctly identify patients who do not develop AKI is limited, particularly as time progresses. The decline in specificity on day 3 may indicate that penKid levels become less reliable for ruling out AKI in the later stages of the postoperative period.

penKid’s was low across all time points (0.23 on day 1 to 0.45 on day 3). This indicates that a proportion of patients with an elevated penKid level may not ultimately develop AKI based on creatinine criteria, highlighting the risk of overestimating risk if the marker is used in isolation. However, the test’s clinical strength lies in its consistently high negative predictive value (NPV), which was 0.93 on day 1 and remained high on subsequent days. This high NPV is clinically significant, as it allows for the confident identification of patients at a genuinely low risk of AKI. This feature enables more efficient allocation of postoperative resources, prevents unnecessary interventions, and allows monitoring to be focused on higher-risk patients.

Numerous studies have demonstrated that PENK can detect AKI with sensitivity comparable to or superior to that of other established biomarkers, such as Neutrophil gelatinase-associated lipocalin (NGAL) and Tissue inhibitor of metalloproteinases 2 (TIMP-2) × Insulin-like growth factor-binding protein 7 (IGFBP-7). This makes it a valuable nephron injury biomarker (Marino et al. 2015; Gayat et al. 2018). Research suggests that PENK levels can reflect glomerular dysfunction and predict new-onset AKI, making it a potential game changer in managing kidney-related complications in intensive care (Schulte 2024) and postoperative settings (Gombert et al. 2020).

In critically ill patients, particularly those undergoing continuous renal replacement therapy, penKid levels have been associated with successful dialysis liberation (von Groote et al. 2022; von Groote et al. 2023; Tichy et al. 2024). Furthermore, the fact that hemodialysis calls do not remove PENK (Jakubowska et al. 2024) suggests that penKid serves as a diagnostic tool and may also provide prognostic insights regarding recovery from AKI.

### Prediction of AKI

The variation in penKid’s accuracy indicates that the predictive performance of penKid remains relatively consistent in the early postoperative period, albeit with moderate accuracy. The higher values of NIR than the observed accuracy at each time point suggest that the prediction of AKI purely based on penKid levels does not exceed the expected performance from random guessing, as reflected in the *p*-values (1.000 for days 1 and 2 and 0.82 for day 3). Therefore, while penKid showed some predictive potential, it did not significantly outperform chance in this cohort.

The low Gwet’s AC1 values suggest that the agreement between penKid predictions and the observed AKI status is limited, and that corrections for chance agreement do not significantly improve the interpretation of proenkephalin’s predictive ability. Moreover, McNemar’s test yielded statistically significant *p*-values on days 1 and 2 (both < 0.001), indicating substantial differences between the predicted and observed classifications, with a trend toward systematic prediction errors. On day 3, however, McNemar’s test *p*-value was 0.061, suggesting that by this time point, the systematic discrepancies between predictions and observed outcomes were less pronounced but still present.

### Detection of subclinical AKI

The prevalence and detection rate results suggest that penKid becomes progressively more effective at identifying sAKI cases as the postoperative period advances. However, the detection prevalence (the proportion predicted to have AKI) was higher than the actual prevalence at all time points, reaching 0.65 on day 3, again indicating a potential overestimation of AKI risk by penKid. The balanced accuracy, which accounts for class imbalances by averaging sensitivity and specificity, remained moderate across all time points, with values of 0.65 on day 1, 0.63 on day 2, and 0.59 on day 3. This suggests that while penKid has some predictive value, it does not offer consistently strong performance, particularly as specificity declines over time.

The use of penKid as a biomarker for predicting sAKI in the early postoperative period showed promise, particularly in its high sensitivity and negative predictive value, which made it a valuable tool for identifying patients at low risk for AKI. However, the moderate accuracy, low specificity, and low PPV suggest that penKid should be used cautiously when predicting sAKI occurrence, particularly in light of its tendency to overestimate AKI risk.

### Pathophysiological rationale for PENK’s diagnostic accuracy

The robust performance of PENK as a biomarker for GFR and AKI is grounded in its physiological characteristics. PENK is a stable fragment derived from the precursor molecule proenkephalin A. Due to its small size and the fact that it is not bound to plasma proteins, PENK is freely and exclusively filtered by the renal glomeruli. Consequently, its plasma concentration is inversely correlated with the GFR. When GFR declines, as in AKI, the filtration of PENK is reduced, leading to its accumulation in the blood (Khorashadi et al. 2020).

The kidneys possess the highest density of delta opioid receptors outside of the central nervous system, and enkephalins, the biologically active peptides for which PENK is a stable surrogate, are thought to have a regulatory effect on renal function, including inducing diuresis and natriuresis. The elevated penKid levels observed during AKI could therefore result from both a decreased glomerular filtration and a simultaneous increase in the production of enkephalins as part of a systemic stress response or a pathophysiological signalling process (Khorashadi et al. 2020).

### PenKid as a part of AKI care bundles

While penKid has the potential to facilitate the early detection of AKI, particularly within the first 48 h after surgery, its use should be considered complementary rather than as a standalone solution. AKI care bundles are excellent examples of comprehensive management of this syndrome, using a structured approach and supplemental biomarkers like penKid. Care bundles have proven to reduce moderate to severe AKI (Schaubroeck et al. 2021). Considering penKid’s predictive value, its limitations must be acknowledged to avoid overdiagnosis and unnecessary interventions. To evaluate the final penKid’s feasibility in perioperative and intensive care settings, an investigation in RCT trials, preferably with a structured AKI bundle, is necessary.

### Limitations and strengths of the study

This study has several limitations. First, we acknowledge the relatively small sample size, which is common in studies of highly specialized procedures and limits statistical power. However, the homogeneity of the cohort, with all procedures and perioperative care standardized and performed by a single team, helped to mitigate the limitations. Second, a generic PENK ELISA kit was used as the only standardized one which was available to our team. Our investigation included a possible sampling bias within the investigated population, as we did not measure with both methods. Finally, our diagnostic criteria for AKI did not include urine output. However, a previous study in a smaller EVAR population reported AKI occurrence using precise urine output recording with almost identical incidence (Saratzis et al. 2015).

More importantly, our data suggest that bedside tests such as penKid are reliable tools for identifying patients with low-risk AKI in a perioperative setting. The cohort was homogeneous, and perioperative management followed a uniformly applied standardized operating protocol. Additionally, we confirmed the superiority of bedside tests versus PENK/ELISA in AKI detection. Future studies could further evaluate the prognostic importance of changes in PENK levels in noncardiac surgery settings.

## Conclusions

penKid shows promise as an early biomarker for risk stratification following complex EVAR. While its low PPV and moderate overall accuracy suggest it should not be used as a standalone diagnostic test, its high sensitivity and NPV make it a valuable tool for ruling out AKI in low-risk patients. Its primary utility lies in its potential integration into structured AKI care bundles, where its early signal can complement clinical assessment and guide proactive management. Although penKid does not fundamentally alter AKI treatment, it supports a more personalized and timely approach.

## Supplementary Information

Supplementary Material: Appendix 1. Packages.

## Data Availability

The complete dataset used in this study has been deposited in the Zenodo repository (10.5281/zenodo.14827489). This dataset is freely available for research purposes under a CC-BY 4.0 license.

## Abbreviations

AC1: Gwet’s AC1 agreement coefficient
AKI: Acute kidney injury
BMI: Body mass index
CI: Confidence interval
ELISA: Enzyme-linked immunosorbent assay
EVAR: Endovascular aneurysm repair
IGFBP-7: Insulin-like growth factor-binding protein 7
IQR: Interquartile range
KDIGO: Kidney Disease Improving Global Outcomes
NGAL: Neutrophil gelatinase-associated lipocalin
NIR: No information rate
NPV: Negative predictive value
PENK: Proenkephalin A 119–159
POC: Point of care
PPV: Positive predictive value
RCT: Randomized controlled trial
RRT: Renal replacement therapy
SAPS: Simplified Acute Physiology Score
SCr: Serum creatinine
sAKI: Subclinical acute kidney injury
SOFA: Sequential Organ Failure Assessment
TIMP-2: Tissue inhibitor of metalloproteinases 2
