# Supplementary material for: Proenkephalin A 119–159 as an early biomarker of acute kidney injury in complex endovascular aortic repair: an explorative single-center cross-sectional study with the utilization of two measurement methods
Source: Perioper Med (Lond). 2025 Jul 4;14:66. doi: 10.1186/s13741-025-00553-5 (PMC12228180; doi:10.1186/s13741-025-00553-5)
Supplement: Supplementary file 1 — Supplementary Material: Appendix 1. Packages. [file 13741_2025_553_MOESM1_ESM.docx]

The packages: rio (version 1.2.1; Chan C et al., 2023), doParallel (version 1.0.17; Corporation M, Weston S, 2022), rngtools (version 1.5.2; Gaujoux R, 2021), doRNG (version 1.8.6; Gaujoux R, 2023), irrCAC (version 1.0; Gwet KL, Ph.(2019), sjPlot (version 2.8.15; Lüdecke D, 2023), parameters (version 0.22.2; Lüdecke D et al., 2020), performance (version 0.12.3; Lüdecke D et al., 2021), report (version 0.5.8; Makowski D et al., 2023), correlation (version 0.8.5; Makowski D et al., 2022), ggstatsplot (version 0.12.3; Patil I, 2021), pROC (version 1.18.5; Robin X et al., 2011), gtsummary (version 1.7.2; Sjoberg D et al., 2021), cutpointr (version 1.1.2; Thiele C, Hirschfeld G, 2021), MASS (version 7.3.60.0.1; Venables WN, Ripley BD, 2002), ggplot2 (version 3.5.0; WickhamH, 2016), dplyr (version 1.1.4; Wickham H et al., 2023).

Chan C, Leeper T, Becker J, Schoch D (2023). rio: A Swiss-army knife for data file I/O. <https://cran.r-project.org/package=rio>.

Corporation M, Weston S (2022). doParallel: Foreach Parallel Adaptor for the 'parallel' Package. R package version 1.0.17, <https://CRAN.R-project.org/package=doParallel>.

Gaujoux R (2021). rngtools: Utility Functions for Working with Random Number Generators. R package version 1.5.2, <https://CRAN.R-project.org/package=rngtools>.

Gaujoux R (2023). doRNG: Generic Reproducible Parallel Backend for 'foreach' Loops. R package version 1.8.6, <https://CRAN.R-project.org/package=doRNG>.

Gwet KL, Ph.D. (2019). irrCAC: Computing Chance-Corrected Agreement Coefficients (CAC). R package version 1.0, <https://CRAN.R-project.org/package=irrCAC>.

Lüdecke D (2023). sjPlot: Data Visualization for Statistics in Social Science. R package version 2.8.15, <https://CRAN.R-project.org/package=sjPlot>.

Lüdecke D, Ben-Shachar M, Patil I, Makowski D (2020). “Extracting, Computing and Exploring the Parameters of Statistical Models using R.” Journal of Open Source Software, 5(53), 2445. doi:10.21105/joss.02445

Lüdecke D, Ben-Shachar M, Patil I, Waggoner P, Makowski D (2021). “performance: An R Package for Assessment, Comparison and Testing of Statistical Models.” Journal of Open Source Software, 6(60), 3139. doi:10.21105/joss.03139

Makowski D, Lüdecke D, Patil I, Thériault R, Ben-Shachar M, Wiernik B (2023). “Automated Results Reporting as a Practical Tool to Improve Reproducibility and Methodological Best Practices Adoption.” CRAN. <https://easystats.github.io/report/>.

Makowski D, Wiernik B, Patil I, Lüdecke D, Ben-Shachar M (2022). “correlation: Methods for Correlation Analysis.” Version 0.8.3, <https://CRAN.R-project.org/package=correlation>.

Makowski D, Ben-Shachar M, Patil I, Lüdecke D (2020). “Methods and Algorithms for Correlation Analysis in R.” Journal of Open Source Software, 5(51), 2306. doi:10.21105/joss.02306 <https://doi.org/10.21105/joss.02306>,

Patil I (2021). “Visualizations with statistical details: The 'ggstatsplot' approach.” Journal of Open Source Software, 6(61), 3167. doi:10.21105/joss.03167

R Core Team (2024). R: A Language and Environment for Statistical Computing. R

Foundation for Statistical Computing, Vienna, Austria. <https://www.R-project.org/>.

Robin X, Turck N, Hainard A, Tiberti N, Lisacek F, Sanchez J, Müller M (2011). “pROC: an open-source package for R and S+ to analyze and compare ROC curves.” BMC Bioinformatics, 12, 77.

Sjoberg D, Whiting K, Curry M, Lavery J, Larmarange J (2021). “Reproducible Summary Tables with the gtsummary Package.” The R Journal, 13, 570-580. doi:10.32614/RJ-2021-053

Thiele C, Hirschfeld G (2021). “cutpointr: Improved Estimation and Validation of Optimal Cutpoints in R.” Journal of Statistical Software, 98(11), 1-27. doi:10.18637/jss.v098.i11

Venables WN, Ripley BD (2002). Modern Applied Statistics with S, Fourth edition. Springer, New York. ISBN 0-387-95457-0

Wickham H (2016). ggplot2: Elegant Graphics for Data Analysis. Springer-Verlag New York. ISBN 978-3-319-24277-4, <https://ggplot2.tidyverse.org>.

Wickham H, Bryan J (2023). readxl: Read Excel Files. R package version 1.4.3, <https://CRAN.R-project.org/package=readxl>.

Wickham H, François R, Henry L, Müller K, Vaughan D (2023). dplyr: A Grammar of Data Manipulation. R package version 1.1.4, <https://CRAN.R-project.org/package=dplyr>.
